# Effects of Neuromuscular Training on Agility Performance in Elite Soccer Players

**DOI:** 10.3389/fphys.2019.00947

**Published:** 2019-07-23

**Authors:** Hassane Zouhal, Abderraouf B. Abderrahman, Gregory Dupont, Pablo Truptin, Régis Le Bris, Erwan Le Postec, Zouita Sghaeir, Matt Brughelli, Urs Granacher, Benoit Bideau

**Affiliations:** ^1^Univ Rennes, M2S (Laboratoire Mouvement, Sport, Santé) – EA 1274, Rennes, France; ^2^ISSEP Ksar Said, University of Manouba, Tunis, Tunisia; ^3^Fédération Française de Football (FFF), Paris, France; ^4^Lorient Football Club (FCL), Lorient, France & Stade Rennais Football Club (SRFC), Rennes, France; ^5^Sports Performance Research Institute New Zealand (SPRINZ), AUT Millennium, Auckland University of Technology, Auckland, New Zealand; ^6^Division of Training and Movement Science, University of Potsdam, Potsdam, Germany

**Keywords:** laterality, football, footedness, eyedness, rotation, team sport

## Abstract

**Background:**

Agility in general and change-of-direction speed (CoD) in particular represent important performance determinants in elite soccer.

**Objectives:**

The objectives of this study were to determine the effects of a 6-week neuromuscular training program on agility performance, and to determine differences in movement times between the slower and faster turning directions in elite soccer players.

**Materials and Methods:**

Twenty male elite soccer players from the Stade Rennais Football Club (Ligue 1, France) participated in this study. The players were randomly assigned to a neuromuscular training group (NTG, *n* = 10) or an active control (CG, *n* = 10) according to their playing position. NTG participated in a 6-week, twice per week neuromuscular training program that included CoD, plyometric and dynamic stability exercises. Neuromuscular training replaced the regular warm-up program. Each training session lasted 30 min. CG continued their regular training program. Training volume was similar between groups. Before and after the intervention, the two groups performed a reactive agility test that included 180° left and right body rotations followed by a 5-m linear sprint. The weak side was defined as the left/right turning direction that produced slower overall movement times (MT). Reaction time (RT) was assessed and defined as the time from the first appearance of a visual stimulus until the athlete’s first movement. MT corresponded to the time from the first movement until the athlete reached the arrival gate (5 m distance).

**Results:**

No significant between-group baseline differences were observed for RT or MT. Significant group x time interactions were found for MT (*p* = 0.012, effect size = 0.332, small) for the slower and faster directions (*p* = 0.011, effect size = 0.627, moderate). Significant pre-to post improvements in MT were observed for NTG but not CG (*p* = 0.011, effect size = 0.877, moderate). For NTG, *post hoc* analyses revealed significant MT improvements for the slower (*p* = 0.012, effect size = 0.897, moderate) and faster directions (*p* = 0.017, effect size = 0.968, moderate).

**Conclusion:**

Our results illustrate that 6 weeks of neuromuscular training with two sessions per week included in the warm-up program, significantly enhanced agility performance in elite soccer players. Moreover, improvements were found on both sides during body rotations. Thus, practitioners are advised to focus their training programs on both turning directions.

## Introduction

In soccer, players are required to accelerate, decelerate, and change direction quickly throughout the game. Bloomfield et al. (2007) observed a mean of 727 body rotations and changes of direction per game and per player in the English Premier League. Moreover, soccer players performed between 1000 and 1400 movements of 2 to 4 s in duration (Bangsbo, 1997; Mohr et al., 2003; Stolen et al., 2005). In field sports like soccer, agility represents an important performance determinant that should be included in the training regime of young players (Davids et al., 2000; Young and Willey, 2010). Agility is an essential performance determinant in soccer and is composed of perceptual and decision-making factors like visual scanning, anticipation, etc. Moreover, agility has traditionally been thought of as simply the ability to change direction quickly. However, more than 30 years ago, Chelladurai (1976) observed that agility is a complex quality containing temporal and spatial uncertainty (Chelladurai, 1976). Although many practitioners classify agility as any movement involving rapid changes of direction, agility has recently been defined as a rapid change of direction (CoD) in response to a sport-specific stimulus. According to Sheppard and Young (22), agility is a rapid whole-body movement with change of velocity or direction in response to a stimulus. Accordingly, agility contains a CoD component (Chaabene et al., 2018) and a perceptual and decision-making component.

The capacity to quickly change direction while sprinting, also well known as a cutting maneuver or CoD, is an important performance determinant in many team sports (e.g., ice hockey, soccer, handball, basketball, etc.) (Paul et al., 2016). CoD predominately characterizes an athletes’ ability to decelerate in the shortest possible time and to re-accelerate quickly in a new direction (Spiteri et al., 2013; Chaabene et al., 2018). In this context, CoD represents the physical quality of agility while perceptual and decision-making factors constitute the underlying cognitive components of agility (Sheppard and Young, 2005). CoD is multifactorial and includes physical qualities such as straight sprinting speed, reactive strength, concentric strength and power (Sheppard et al., 2006). Furthermore, the technical execution of CoD tasks can also be influenced by the underlying biomechanical specifics such as the angle-velocity trade-off (Dos’Santos et al., 2018).

Jones et al. (2009) computed associations between several physical attributes (e.g., speed, strength and power) and CoD performance in University students. Their results suggested that for CoD improvements, athletes should seek to maximize their sprinting ability and enhance their eccentric knee flexor strength to allow effective neuromuscular control during the contact phase of the CoD task (505 test) (Jones et al., 2009). Previously, many different training protocols have been applied to enhance agility (Polman et al., 2004; Miller et al., 2006; Paul et al., 2016). For instance, Polman et al. (2004) observed significant improvements in the ability to quickly turn left and right after a 12-week CoD training program in soccer players. In another study, Miller et al. (2006) investigated the impact of a 6-week plyometric training program in untrained individuals and observed significant improvements in CoD performance (i.e., Illinois Agility Test). Despite these study findings and in accordance with Sheppard and Young, it seems that single-mode training does not translate to improvements in agility performance (Sheppard and Young, 2005). Hence, Myer et al. (2011) suggested that integrative neuromuscular training incorporating fundamental and specific movements through dynamic stability, balance, core strength, plyometric and agility exercises may improve movement biomechanics and minimize the risk of sustaining injuries.

It has previously been demonstrated that during CoD tasks to one side, faster athletes tend to have better strength in the leg responsible for the push-off action (Young et al., 2002). Of note, better static balance and reactive strength were observed in the support leg (e.g., non-dominant leg) (Carey et al., 2001; Valdez, 2003). There is evidence that soccer players kick the ball faster and with more accuracy with their dominant leg (Carey et al., 2001; Valdez, 2003). However, other studies reported only small advantages of the dominant leg when it comes to kicking (Kramer and Balsor, 1990; McLean and Tumilty, 1993; Mognoni et al., 1994). In elite soccer players, Bloomfield et al. (2007) clearly showed that the ability to rotate the body quickly represents an important match performance determinant. In fact, these authors recorded more than 700 body rotations and turns during a single match (Bloomfield et al., 2007). Among the overall number of body rotations during a match, 300 were performed from 0 to 180°. These actions often take place during a match if players aim to evade an opponent after controlling the ball (Bloomfield et al., 2007). Thus, footedness appears to play a crucial role for performance during body rotations (e.g., CoD component of agility) (Zouhal et al., 2019). Previous research has indicated that there is a laterality effect in athletes when they turn quickly (Azémar et al., 2008; Zouhal et al., 2018). Recently, Zouhal et al. (2018) demonstrated a laterality effect in elite soccer players when performing 180° left and right body rotations. These authors revealed better performances if the players used their supporting leg for the initiation of body rotations. In addition, the analysis indicated better performances if right-footed players performed quick turns that were initiated by their left leg. From these findings, the authors concluded that each player had “slower” and “faster” turning direction. Interestingly, Polman et al. (2004) observed different turning patterns during an agility test in soccer players. However, coaches demand that their players can turn similarly on either side in order to improve performance (Young et al., 2002; Polman et al., 2004).

Consequently, the main purpose of this study was to examine the effects of a neuromuscular training program on agility performance and to determine differences in movement times between the slower and faster turning directions in elite soccer players. In addition, we aimed to reduce potential laterality effects during quick left and right turns in the examined cohort. With reference to the relevant literature (Sheppard and Young, 2005; Myer et al., 2011; Granacher et al., 2016; Zouhal et al., 2018), the main hypotheses of the current study were that (i) neuromuscular training will enhance agility performance and (ii) this type of training diminishes potential laterality effects of soccer players.

## Materials and Methods

### Participants

Twenty male elite soccer players (6 defenders, 8 mid-fielders and 6 strikers) from the Stade Rennais Football Club (Ligue 1 represents the first professional soccer division in France) participated in this study (Table 1). On average, the players practice 10–11 months per year with 5–6 training sessions and one match per week. According to playing position, they were randomly assigned to a neuromuscular training group (NTG, *n* = 10) or a control group (CG, *n* = 10). Each group included 3 defenders, 4 mid-fielders and 3 strikers.

**TABLE 1:** Anthropometric parameters of the soccer players.

**Soccer players**	**Age (yrs)**	**Height (cm)**	**Mass (kg)**	**Fat mass (%)**
NTG (*n* = 10)	17.7 ± 0.4	178.0 ± 8.7	69.1 ± 7.6	9.6 ± 1.4
CG (*n* = 10)	16.8 ± 0.7	179.5 ± 7.8	66.6 ± 9.7	9.4 ± 1.6

*Data are means and standard deviations. NTG, Neuromuscular training group; CG, control group.*

**FIGURE 1:**
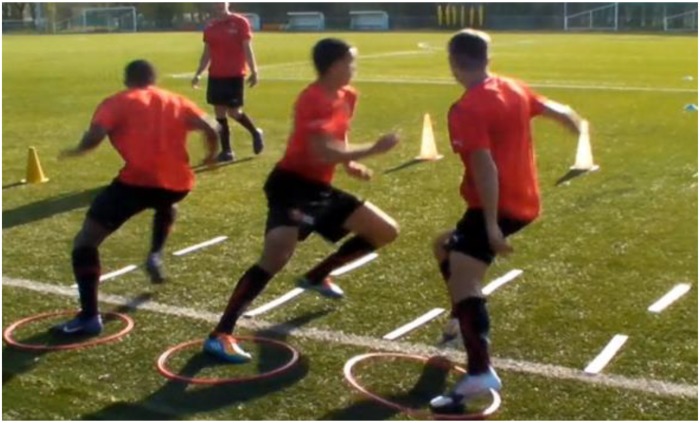
Example of exercise training to learn motor rotation.

**FIGURE 2:**
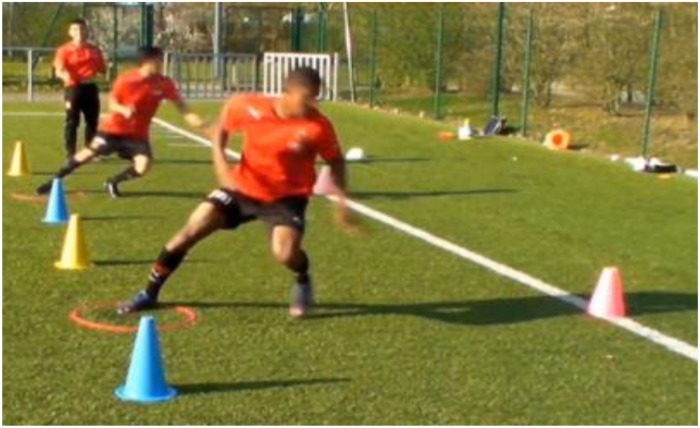
Example of exercise training to train general rotation.

**FIGURE 3:**
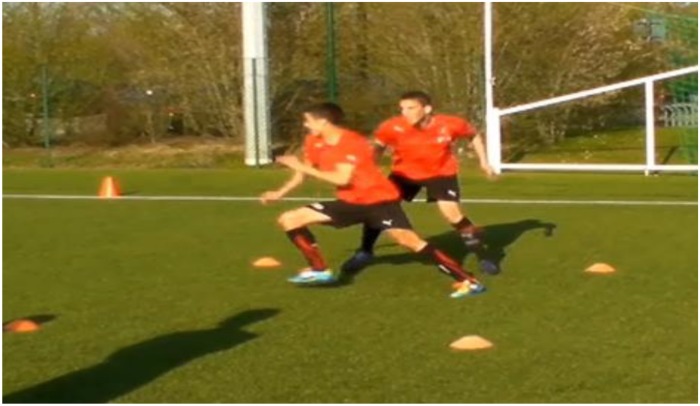
Example of exercise training for a duel between two players to train change of direction (CoD).

**FIGURE 4:**
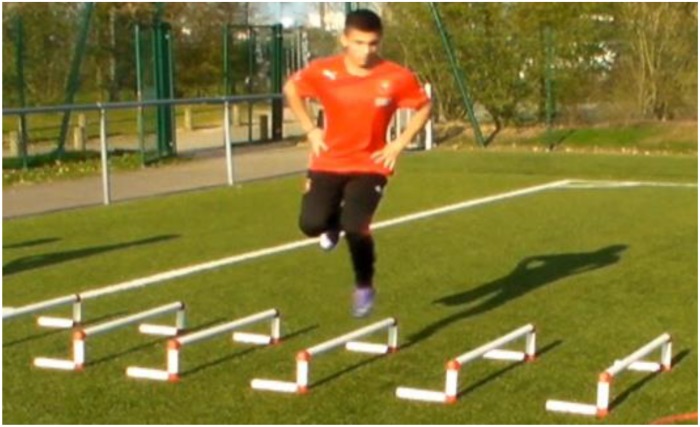
Example of exercise for plyometric training.

Before the study started, written informed consent was obtained from each participant. Our study was conducted in accordance with the international ethical standards. The Ethical Committee on Human Research of the University of Rennes 2, France, approved the study and it was in accordance with the latest version of the Declaration of Helsinki. Moreover, another written informed consent was obtained from all players appearing in Figures 1–4.

An *a priori* power analysis [expected SD of residuals = 50 ms for reaction time (RT) and 0.05 s for movement time (MT), desired power = 0.80, and alpha error = 0.05] was computed using a related study (Zouhal et al., 2018) to simulate a statistically significant group by time interaction effect for our primary outcome (i.e., agility performance). The analysis revealed a minimum sample size of *n* = 7 per group.

### Procedures

Before the start of our experimental protocol, participants were habituated with all testing equipment and procedures. To allow sufficient recovery, the last training session was conducted 48 h prior to testing. To minimize any effects of diurnal variation, the two testing sessions were conducted within 2 h of the same time of day. Players were instructed to wear the same footwear for all sessions.

Before the start of the study, eyedness, handedness, and footedness were determined to establish the soccer player’ laterality profile (Azémar et al., 2008; Zouhal et al., 2018). In other words, we assessed the dominant eye, hand, and foot through a well-established questionnaire (Azémar et al., 2008).

Pre-and-post tests included an agility test as previously described by Zouhal et al. (2018). Briefly, the test consisted of 180° body rotations in response to visual stimuli which indicated the side of the rotation. The player started in an upright standing position with their feet placed in a parallel position. Two meters in front of the athlete was an electronic device, which displayed the visual stimulus, and 5 m behind the athlete was an arrival gate. Application software Virtools generated real-time 3D visual lights (Virtools Software Suite 3.5, Copyright© 2006 Dassault Systemes, France). The lights were composed of three balls of green colors modeled in 3D. The balls were similar in size and lighted with the same intensity. The device showed the side of the rotation: when the left light turned red, the player performed a left rotation and conversely when the right light turned red, the player performed a right rotation. When the center light turned red, the player was able to individually select the preferred side to turn. The device was fixed on a tripod with adjustable height. Thus, each test was performed at the players’ eye level. To perform the agility test, players had to execute 180° turns to the left or right side and accelerate quickly to the arrival gate which was placed 5 m behind the athlete. Each player had to execute the test nine times: three times for each light in randomized order due to the software: left, right or center rotation. Before the test started, each player performed a 20 min standardized warm-up. A one-minute rest was allowed between test trials. After the initial familiarization session and in order to assess test-retest reliability, all players performed the test two times on two different test days separated by at least 2 days. There after, participants in the NTG followed a 6-week training program with two sessions per week in addition to their regular training with the team. After the intervention period, baseline tests were repeated.

### Collecting Data

As in the study of Zouhal et al. (2019), participants’ reaction times (RT) were obtained using an accelerometer, which was attached to the players’ chest and connected via Bluetooth to a computer. It recorded the time between signal appearance and the beginning of the player’s movement (e.g., first foot’s movement detected). The participants’ movement times (MT) were collected using four cameras (SANYO HD) that were placed around the test area (Zouhal et al., 2019).

### Neuromuscular Training

During the intervention period, experienced coaches and sport scientists trained NTG and CG. Only NTG received the neuromuscular training program over a 6-week in-season period with two sessions per week including agility, stability and plyometric exercises. Each session lasted 30 min and neuromuscular training replaced the regular warm-up program conducted by CG. CG continued their regular training routine, which included a 30 min warm-up consisting of running and dribbling drills, passes and goal scoring exercises. Training volumes were similar between the two groups. All soccer players were free of injuries during training and testing.

Neuromuscular training group conducted a 5 to 10 min general warm-up program consisting of running and dribbling drills. Neuromuscular exercises were conducted on the pitch and in the gym. While players performed agility, CoD and plyometric exercises on the pitch, dynamic stability, balance and core strength exercises were conducted in the gym.

On the pitch, the first exercise was devoted to learn how to turn quickly and how to initiate the body rotation. For this purpose, hoops were used to train players where to put their first press-hold, and then corridors were formed with slats to make them turn in the right direction (Figure 1). In fact, it is well known that a good placement of the support leg could be the best way to teach players how to return, in order to optimize CoD performance. In addition, to stimulate and promote fast reaction times (RT), players had to react to different signal types [e.g., auditory cues (beep), visual cues (different colors of cones)] during exercise.

Then, the second exercise was used to train general body rotations (Figure 2). For this purpose, players performed many repetitions to the left and right side. In this exercise, the player focused especially on the position of his body segments, in order to choose the best one to start the movement. And finally, the last exercise was based on a soccer specific CoD task (Figure 3). This exercise included rotations that were similar to match situations. The player 1, who was marked by player 2, having to change direction and get around player 2 who was trying to catch him before crossing the finish line. In this exercise, player 2 gave the departure signal. All exercises were performed on the pitch and followed fundamental principles of sport science such as specificity, overload, and progression during training. In other words, the selected exercises were specific but not similar to the tested tasks. Training intensity (e.g., number of repetitions) and training volume (number of foot contacts) were progressively increased over the course of the study period.

For plyometric training, the intensity was light and the focus was on the application of horizontal force during the CoD (Figure 4). Our training program is in accordance with a previous study from Miller et al. (2006). The training volume ranged from 40 to 60 foot contacts per session while the intensity of the exercises increased constantly. Training was supervised and players received instructions on how to perform each exercise. The exercises included side to side ankle hops, standing long jumps, one-legged lateral jumps, lateral jumps over a hurdle, double leg hops, cone hops with 180° side-cuts.

In the gym, after each training session, NTG performed dynamic stability, core strength and balance exercises. For this purpose, 4 to 5 exercises were conducted using Swiss-balls, elastic band straps, BOSU balls and inflated disks. The task difficulty increased progressively according to the progress of each player. When the player accomplished an exercise, task difficulty was increased. During this time, CG performed their regular stretching exercises.

In addition, a focus during training was on exercising the slower turning direction to diminish the laterality effect. In other words, the training program was equally distributed to exercise both, slower and faster directions. Coaches and sport scientists supervised all training sessions.

### Statistical Analyses

Data are presented as means and standard deviations (SD). After normality of data distribution was confirmed using the Shapiro-Wilk test, differences within and between groups were calculated using a two-way analysis of variance (ANOVA) for repeated measures. If group x time interactions turned out to be significant, a Newman-Keul’s *post hoc* test was calculated. Additionally, effect sizes (ES) were determined from ANOVA output by converting partial eta-squared to Cohen’s d. Moreover, within-group ES were computed using the following equation: ES = (mean post – mean pre)/SD (Cohen, 1988). In accordance with Hopkins et al. (2009) ES were considered trivial (<0.2), small (0.2–0.6), moderate (0.6–1.2), large (1.2–2.0) and very large (2.0–4.0). The level of significance was set at *p* < 0.05. All statistical analyses were computed using SPSS for Windows, version 16.0 (SPSS Inc., Chicago).

## Results

All players from both experimental groups completed the study according to the previously described study design and methodology. No injuries related to training or testing occurred over the course of the experimental period. During the 6-week intervention period, attendance rates amounted to 93.2% for NTG and 91.7% for CG.

High test-retest reliability was found for RT (*r* = 0.899) and MT (*r* = 0.913). In addition, intraclass correlation coefficients revealed an ICC of 0.735 for RT and an ICC of 0.879 for MT. These ICC values were almost perfect according to the classification of Landis and Koch (1977).

No significant between-group baseline differences were found for any of the analyzed parameters (Tables 2–4).

**TABLE 2:** Reaction time performances determined during the Agility test before (pre test) and after (post test) the training program for control group (CG) and training group (NTG).

			***p*-value (ES)**		
**Reaction Time (s)**	**CG (*n* = 10)**	**NTG (*n* = 10)**	**Time**	**Group**	**Group × Time**
Pre Test	0.381 ± 0.025	0.385 ± 0.030	0.331 ± 0.357	0.363 (0.258)	0.453 (0.278)
Post test	0.389 ± 0.024	0.365 ± 0.020	0.178 ± 0.269	0.479 (0.398)	0.275 (0.353)

*Data are mean and standard deviations. ES, Effect size; MT, Movement time; NTG, Neuromuscular training group; CG, Control group.*

**TABLE 3:** Movement time performances determined during the Agility test before (pre test) and after (post test) the training program for control group (CG) and training group (NTG).

			***p*-value (ES)**		
**Movement Time (s)**	**CG (*n* = 10)**	**NTG (*n* = 10)**	**Time**	**Group**	**Group × Time**
Pre Test	1.468 ± 0.07	1.451 ± 0.08	0.156 (0.399)	0.553 (0.278)	0.165 (0.138)
Post test	1.444 ± 0.06	1.383 ± 0.07^*^	0.011 (0.971)	0.017 (0.918)^$^	0.012 (0.332)

Data are mean ± SD. ES, Effect size; MT, Movement time; NTG, Neuromuscular training group; CG, Control group. ^*^: Significant difference between Pre and Post, ^*^p < 0.05. $: Significant difference between CG and NTG, ^$^*p* < 0.05.

**TABLE 4:** Differences between the weak and strong side in movement time (MT) for the neuromuscular training group (NTG) and control group (CG).

		**CG (*n* = 10)**		**NTG (*n* = 10)**		***p*-values (ES)**		
		**Slower direction**	**Faster direction**	**Slower direction**	**Faster direction**	**Time**	**Group**	**Group × Time**
	MT Pre Test (s)	1.456 ± 0.05	1.460 ± 0.08	1.461 ± 0.09	1.434 ± 0.07	**Slower direction** 0.236 (0.424)	**Slower direction**0.191 (0.143)	**Slower direction** 0.092 (0.189)
						**Faster direction** 0.343 (0.559)	**Faster direction** 0.251 (0.371)	**Faster direction** 0.765 (0.201)
	MT Post Test (s)	1.431 ± 0.06	1.421 ± 0.05	1.388 ± 0.05	1.374 ± 0.06	**Slower direction** 0.012 (0.898)^*^	**Slower direction** 0.023 (0.715)£	**Slower direction** 0.011 (0.627)£
						**Faster direction** 0.017 (0.968)^*^	**Faster direction** 0.019 (0.945)£	**Faster direction** 0.021 (0.897)**£**

Data are means and standard deviations. ES, Effect size; MT, Movement time; NTG, Neuromuscular training group; CG, Control group. ^*^: Significant difference between Pre and Post, ^*^p < 0.05. £: Significant difference between CG and NTG, ^£^*p* < 0.05.

Performances in MT during the agility test are presented in Table 3. A significant group × time interaction was found for MT performances (*p* = 0.012, effect size = 0.332, small). *Post hoc* tests revealed significant pre-to post improvements for NTG but not CG (*p* = 0.011, effect size = 0.971, moderate).

Table 4 illustrates findings for MT according to the slower and faster direction no significant differences were observed between MT performances of the slower and faster direction at baseline neither within and between groups.

Significant group × time interactions were found for MT performances on the slower direction (*p* = 0.011, effect size = 0.627, moderate) and for the faster direction (*p* = 0.021, effect size = 0.897, moderate). For NTG, significant pre-to-post improvements were found for MT performance on the slower (*p* = 0.012, effect size = 0.897, moderate) and the faster turning sides (*p* = 0.017, effect size = 0.968, moderate).

## Discussion

To our knowledge, this was the first study that examined the effects of neuromuscular training on the ability to perform 180° body rotations in elite soccer players. The main findings of this study were that (i) 6 weeks of neuromuscular training with 2 sessions per week significantly improved turning performance (180°) in elite soccer players and (ii) this neuromuscular training program was able to enhance performance on the slower turning side in trained players.

Laterality is responsible for functional asymmetries during several actions as turning (rotations). It is necessary to differentiate the leg used to hit or manipulate objects (called the “dominant” leg) and the leg responsible for supporting the posture and the balance in the thrust and rotation movements (called the support leg). When right-handers turn to the left they use their supporting leg, this gives a better result than when they turn to the right where they use their dominant leg. The effect is even more pronounced in footballers because they have a sporting habit of excessively using their supporting leg during a pass, hit, jump or reception (Carey et al., 2001; Golomer and Mbongo, 2004; Barone et al., 2010). It is well known that soccer player have a foot to strike, and a foot to support jumps or rotations (Lacour et al., 1974; Azémar et al., 2008). The laterality of the footballer is defined by his/her preferential foot (or dominant) for the ball and the dribble (Carey et al., 2001). However, very high-level players use both feet with the same efficiency (Porac and Coren, 1981; Carey et al., 2001). The influence of laterality on a 180-degree turning movement, with a special reference to leg dominance, was studied recently (Zouhal et al., 2018). The main findings showed that the reaction times depended on the side of the visual stimulus. Hence, their results demonstrated that the movement times depended on the side of the rotation and appeared to be faster when the player turned on the contralateral side with their dominant foot. These results indicated that a soccer player has a weakness in his ability to turn due to lateralization, and the authors suggested the importance to train the slower turning direction of the player.

### Effects of Training on Reaction Time

Little and Williams (2005) reported that agility movements represent about 11% of the total distance traveled during a high level match. More specifically, there is evidence that these movements can influence a match. Agility is an important performance determinant in elite soccer and is also used to identify young talented soccer players (Reilly et al., 2000). Concerning performance on the agility test, results from the current study showed no significant differences for the RT between pre and post-tests for both NTG and CG. After the neuromuscular training program, no significant decrease was found for RT. However, it can be assumed that a longer training period could significantly reduce the RT during the agility test (Lacour et al., 1974; Azémar et al., 2008). The perception-action coupling represents the cognitive determinants of agility. First, the visual, auditory or kinesthetic system detects a stimuli, thereafter the respective sensory information is processed within the central nervous system, and finally decision-making results in an adequate neuromuscular response (Sheppard et al., 2006). However, the cognitive part of agility performance is minor because it lasts only a fraction of a second. The essential determinant is the physical determinant (CoD ability) because it takes up the main part of the entire task.

### Effects of Training on Movement Time Performance

In the current study, MT performances recorded during the agility test showed a significant decrease in NTG only. In addition, a significant difference was observed between CG and NTG after the training program. Before and after the training program no significant differences were observed between the slower and faster direction for the two groups, CG and NTG. After the training program and only for NTG, a significant difference was observed concerning MT between pre and post training for both the slower and faster direction. Moreover, the results also showed that NTG performed significantly better on MT than CG for both the slower and faster directions. These results clearly indicate the effectiveness of the training program to increase the 180-degree turning performance. Linear speed and lower limb strength and power qualities are associated with CoD but not agility performance. Thus, training induced improvements in agility can be achieved through the promotion of CoD and its underlying physical qualities as well as perception and decision making factors. Polman et al. (2004) improved agility of elite football players after a 12-week training program consisting of exercises with one or more CoD. On the other hand, training the reactive force of the lower limbs improves agility. Miller et al. (2006) observed significant improvements in performance on the Illinois test in adult’s athletes after 6-week of plyometric training. Furthermore, Attene et al. (2015) recorded improvements in jump performances after 4 weeks of repeated sprint ability with one CoD in male basketball players. In addition, Brughelli et al. (2008) stated that CoD training performed with a horizontal application of force improves the agility performance while CoD training with vertical application of force does not allow improvement of the agility performance. Performance in agility depends on the ability to respond to a stimulus and the ability to change direction. To improve the performance in agility it is necessary to train these physical factors while taking into account the laterality (e.g., slower and faster direction) of the athlete. Regarding the training set up to improve the speed of rotation and reduce the gap between the slower and the faster direction of the players, several points are to be explained in terms of content of training and duration. On the one hand, at the content level, the exercises were set up after a review of the literature on the physical determinants of CoD. Moreover, these exercises were adapted to the practice of soccer because the goal of the training was to make the training similar to the competition. In fact, according to the concept of training specificity (Behm and Sale, 1993) the greatest transfer effects occur when the training movements most closely resemble the sport activity. Hence, part of the training on learning the ability to turn was the most important. Indeed, there has been awareness on the part of players about their ability to turn faster on one side. Before the training, each player individually watches the videos of his pre-test by explaining the technical defects of their rotations (placement of supports, incomplete rotations…). Thus, the initial sessions of the training were intended to teach the players a new motor skill. The second part of the training was in the continuity of learning the ability to turn. Indeed, we have implemented exercises based on the repetition of movement to make the execution of the latter automatic. The third and final part of the training was dedicated to putting into practice this specific ability to turn in match conditions. We put players in a match situation involving rotations (example of the attacking duel *vs.* defender). In parallel with this progress on the automatic rotation, plyometrics allowed the players to improve their pushing force during the supports and thus reduce their ground contact time, which is a determinant of agility (Sheppard et al., 2006). In summary, the content of our training followed logic of learning to move toward the real conditions of a match, which is relevant to the objective of improving performance.

A limitation of this study was that we did not assess linear speed as well as lower limb strength and power. This may have helped to better explain training-induced increases in MT. Moreover, the applied agility test has not yet been used in correlational analyses to examine whether there were associations between agility test performance and relevant match-related key performance indicators in soccer that were related with overall soccer performance. Given the diverse physiological, technical, and tactical demands in elite soccer and the constantly changing situations during a game, laterality is not the only factor that determines a players turning direction.

### Practical Applications

The slower and the faster directions of players seemed to influence their agility performance, which is known to be important for soccer performance. Fitness coaches should identify the slower and faster direction of each player to specifically tailor agility training. The focus should be laid on training both, the slower and faster turning directions. For example, this kind of short neuromuscular training program can be incorporated as part of a warm-up.

## Conclusion

Our results showed that only 6 weeks of neuromuscular training program with 2 sessions per week and a duration of 30 min per session significantly increased turning performance (180°) in elite soccer players. Moreover, this training program was able to improve performance to quickly turn to both directions but specifically on the slower direction.

## Data Availability

The datasets generated for this study are available on request to the corresponding author.

## Ethics Statement

The Ethical Committee on Human Research of the University of Rennes 2, France, approved the study and it was in accordance with the latest version of the Declaration of Helsinki. Moreover, another written informed consent was obtained from all players appearing in Figures 1–4.

## Author Contributions

HZ conceived and designed the study. PT, RLB, ELP, ZS, and BB conducted the experiment. PT, GD, ZS, and AA analyzed the data. PT, GD, MB, AA, BB, UG, and HZ wrote the manuscript. All authors read and approved the manuscript.

## Conflict of Interest Statement

RLB and ELP were employed by company Stade Rennais FC and now they are employed by Lorient Football Club (FCL). GD was employed by the company Fédération Française de Football (FFF). The remaining authors declare that the research was conducted in the absence of any commercial or financial relationships that could be construed as a potential conflict of interest.
